# Should we change the treatment plan in early hepatocellular carcinoma with chronic kidney disease?

**DOI:** 10.1186/s12893-023-01983-y

**Published:** 2023-04-11

**Authors:** Wu-Po Chao, Shion-Wei Chai, Po-Hsing Chiang, Ta-Chun Chou, Yi-Chan Chen, Ruey-Shyang Soong

**Affiliations:** 1 Division of General Surgery, Department of Surgery, Keelung Chang Gung Memorial Hospital, No. 222, Maijin Rd., Anle Dist, Keelung City, 204201 Taiwan; 2 Division of General Surgery, Department of Surgery, Wan Fang Hospital, Taipei Medical University, No.111 Sec.3, Xinglong Rd., Wenshan Dist, Taipei City, 116 Taiwan; 3 College of Medicine, Taipei Medical University, 250 Wu-Hsing Street, Taipei City, 110 Taiwan; 4 Division of Transplantation Surgery, Department of Surgery, Wan Fang Hospital, Taipei Medical University, Taipei, Taiwan; 5 TMU Research Center for Organ Transplantation, Taipei Medical University College of Medicine, Medical University, Taipei, Taiwan

**Keywords:** hepatocellular carcinoma, chronic kidney disease, hepatectomy, radiofrequency ablation, survival

## Abstract

**Background:**

Chronic kidney disease (CKD) has been considered to be a poor prognostic factor for hepatocellular carcinoma (HCC). However, few studies have focused on early HCC and the impact of CKD on survival, which should be considered in curative treatment for early HCC.

**Materials and methods:**

Patients with BCLC stage 0/A were enrolled from 2009 to 2019. A total of 383 patients were divided into Control group and CKD group, based on estimated glomerular filtration rate. Overall survival (OS) and disease-free survival (DFS) of different treatments were determined using the Kaplan–Meier method.

**Results:**

The Control group had a significantly better OS than the CKD group (72.6 months vs. 56.7 months; *p* = 0.003). DFS was similar between the groups (62.2 months vs. 63.8 months, *p* = 0.717). In the Control group, the surgically treated (OP) group had significantly superior OS (65.0 months vs. 80.0 months, *p* = 0.014) and DFS (50.9 months vs. 70.2 months, *p* = 0.020) than the radiofrequency ablation-treated group. In the CKD group, the OP group showed a survival advantage in OS (70.6 months vs. 49.2 months, *p* = 0.004), while DFS was similar between treatment groups (56.0 months vs. 62.2 months, *p* = 0.097).

**Conclusion:**

CKD should not be considered to be a poor prognostic factor in early HCC patients. Moreover, hepatectomy should be carried out in CKD patient with early HCC for better prognosis if feasible.

## Introduction

Hepatocellular carcinoma (HCC) is the most common primary cancer of the liver in the modern era and is the second leading cause of cancer death in East Asia [1, 2]. Multiple risk factors have been associated with HCC, including alcohol addiction, non-alcoholic liver disease, cirrhosis, and particularly viral hepatitis, such as hepatitis B (HBV), C (HCV), and D [3].

The management of HCC is multidisciplinary. Ablation, curative liver resection, and liver transplant are considered to be the standard curative treatment for early HCCs (Barcelona Clinic Liver Cancer Classification [BCLC] 0 and A) [4, 5]. Treatment options are based on feasibility, surgical risk, and availability of liver donors. However, radiofrequency ablation (RFA) and surgery are used in the major proportion BCLC stage 0/A cases due to the scanty of liver donors.

Multiple prognostic factors for the treatment of HCC have been identified, including tumor staging, etiologies, liver function, and comorbidities [6]. Among these factors, chronic kidney disease (CKD) has long been considered to be a poor prognostic factor for HCC patients [7, 8]. Some studies have reported a higher post-operative complication rate in HCC patients with CKD than in those with preserved renal function [9] Several studies from medical institutions in Taiwan have shown that HCC patients with renal dysfunction might have an inferior outcome [10, 11]. However, other studies have found an acceptable outcome in non-CKD as well as in CKD patients [9, 12]. In clinical practice, patients sometimes undergo RFA due to impairment of renal function. Evidence for guiding treatment options in early HCC patients with CKD is lacking.

Therefore, we conducted this retrospective study on early-stage HCC patients treated under BCLC guidelines and analyzed the impact of CKD and different interventions (RFA and surgery). This study sought to determine whether CKD is a poor prognostic factor for early HCC patients and whether surgery in early HCC patients with CKD patients provides survival advantages or only increases risk.

## Materials and methods

This study was conducted under the approval of the Institutional Ethics Review Board of Keelung Chang Gung Memorial Hospital (IRB number:191,218,044).

We retrospectively reviewed patients diagnosed with HCC between January 2009 and December 2019. Data were recorded until December 2021. Patients were included if BCLC were stage 0/A, whom all underwent either surgery or RFA.

The application of RFA or operation was based on BCLC stage and performance status after tumor board consensus and shared decision-making with the patient. If the patient met the criteria of RFA, they would undergo RFA by a hepatologist. Patients who were treated surgically underwent liver resection for curative treatment of HCC via either laparoscopic or an open approach.

These patients were followed-up by regular blood tests, tumor markers, and image studies every 3 months. Tumor recurrence was diagnosed when computed tomography or magnetic resonance imaging showed classic features of HCC, as reported by a radiologist.

### Chronic kidney disease classification

**Fig. 1:**
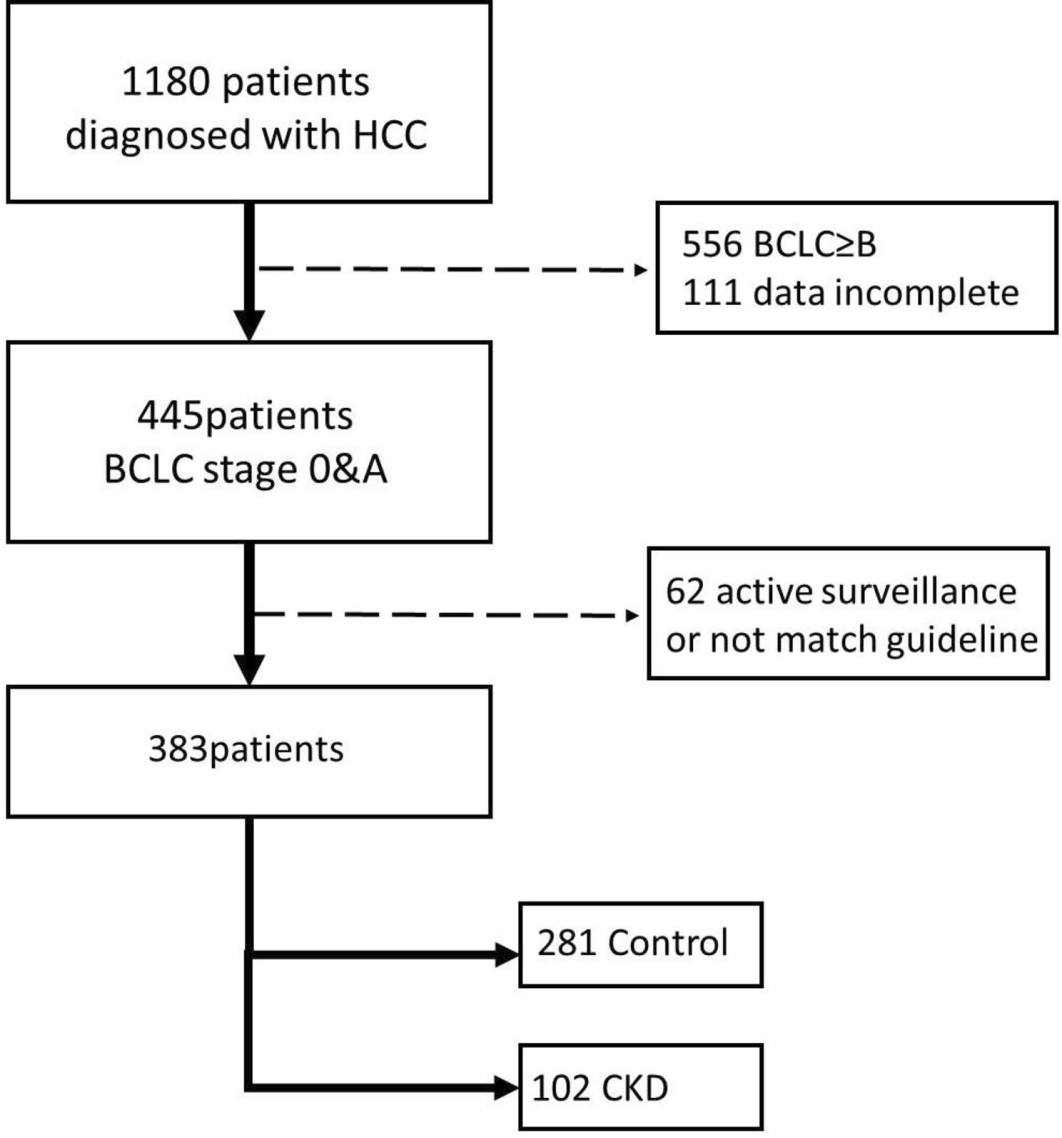
Flowchart of patient selection for this study

In our study, CKD group was defined with eGFR < 60, which was calculated by using the Simplified Modification of Diet in Renal Disease equation [13]. It’s worth noting that eGFR is an estimated value and may not always accurately reflect true kidney function. Additionally, spot eGFR alone is not sufficient for a diagnosis of CKD. The Kidney Disease: Improving Global Outcomes (KDIGO) guidelines recommend obtaining at least two eGFR measurements 90 days apart or performing a urine analysis in those eGFR > 60 to confirm the diagnosis of CKD. However, it is understandable that in some cases, it may not be possible to obtain these additional tests practically. In these cases, using a cut-off value of 60 can be a useful way to identify patients with decreased kidney function. We thus divided the patients into two groups: the Control group with eGFR > 60, and the CKD group, with eGFR < 60, to investigate the effect of kidney dysfunction (Fig. 1).

### Perioperative management for hepatectomy in CKD patient

The application of liver resection criteria and the choice of surgical procedure were made regardless of renal function. We always monitor patients’ volume status with FloTrac™ system perioperatively. Stroke volume variation (SVV) will be maintain above 12%, avoiding overflow in hepatic vein. However, once the liver was transected, adequate IV fluid will be administrated along with fresh plasma based on urine output and adjust with diuretics accordingly. Blood test will be carried on the next day to monitor electrolyte and renal function in case of hyperkalemia and acute kidney injury.

### Statistical analysis

Statistical analyses were performed using SPSS for Windows Ver. 25 (IBM SPSS Inc., Armon, NY, USA). Independent sample *t*-tests were used to compare the differences between the two groups. To overcome selection bias, a propensity score matching (PSM) was performed based on each column listed if group characteristics differed markedly with caliper width of 0.02. However, the PSM was unable to carried out in subgroup analyze due to small sample size. Survival curves, including overall survival (OS) and disease-free survival (DFS) were calculated according to the Kaplan–Meier method and statistical significance was accepted at the *p* < 0.05 level. Cox proportional hazard model was performed in subgroup analyze to estimate hazard ratio in OS and DFS, and statistical significance was accepted at the *p* < 0.05 level.

## Results

There were total 1,180 patients with HCC were extract from the data base. Among these, 445 patients were BCLC stage 0/A. After excluding cases under active surveillance and those with incomplete data, the remaining 383 patients, whom all underwent either surgery or RFA, were enrolled.

### Patient characteristics and treatments

Based on the pre-treatment final renal function evaluation, 383 patients were divided into the Control (n = 281) and CKD (n = 102) groups. The pre-treatment characteristics of these two groups are shown in Table 1. The CKD groups included older patients (64.00 vs. 70.25 years, *p* < 0.001), while the Control groups had a greater proportion of patients with a history of alcohol consumption (30% vs. 15%, *p* = 0.002). The Control groups included more HBV carriers (49% vs. 33%, *p* = 0.005), whereas the CKD groups had more HCV carriers (30% vs. 48%, *p* = 0.007). To avoid bias between these two groups, we performed PSM. The results are shown in the PSM column of Table 1. Ninety-eight patients could be matched, with no significant factor differences among the two groups.

**Table 1:** Baseline characteristic of BCLC 0/A hepatocellular carcinoma patients with chronic kidney disease at various stages, who received intervention (n = 383)

Variables	Original Groups			After PSM			
	Control (n = 281)	CKD (n = 102)	*p*-value	Control (n = 98)	CKD (n = 98)		*p*-value
Sex, female, n(%)	90 (32%)	44 (43%)	0.051	40 (41%)	41 (42%)		0.885
Age, year (mean ± SD)	64.00 (10.68)	70.25 (9.53)	< 0.001*	69.01 (10.30)	70.03 (9.54)		0.473
BMI, kg/m^2^ (mean ± SD)	24.9 (4.44)	25.6 (3.72)	0.120	25.26 (5.34)	25.66 (3.73)		0.649
Tumor size (mm)	25.95 (13.95)	25.72 (11.27)	0.866	26.11 (15.1)	25.88 (11.3)		0.902
BCLC stage, A, n (%)	202 (72%)	79 (77%)	0.249	80 (82%)	75 (77%)		0.382
Alcohol consumption	87 (31%)	15 (15%)	0.002*	20 (20%)	15 (15%)		0.354
ECOG PS, n (%)							
0	274 (97.5%)	94 (92%)	0.017*	93 (94.9%)	91 (92.8)		0.554
1	5 (1.8%)	8 (8%)	0.004*	4 (4.1%)	7 (7.2%)		0.354
≥ 2	2 (0.7%)	0	0.079	1 (1.0%)	0		0.319
AFP > 20 ng/ml, n (%)	55 (20%)	15 (15%)	0.139	18 (18%)	15 (15%)		0.569
Child–Pugh Class, n (%)							
A	273 (97%)	97 (95%)	0.193	93 (95%)	93 (95%)		1
B	8 (3%)	5 (5%)	0.193	5 (5%)	5 (5%)		1
HBV, n (%)	138 (49%)	34 (33%)	0.005*	29 (30%)	34 (35%)		0.447
HCV, n (%)	93 (33%)	49 (48%)	0.007*	49 (50%)	47 (48%)		0.776
HBV&HCV co-infection, n (%)	13 (4.6%)	6 (5.9%)	0.637	7 (7%)	6 (6%)		0.775

BMI: Body mass index; ECOG PS: Eastern Cooperative Oncology Group performance status; AFP: Alpha-fetoprotein; HBV: Hepatitis B virus; HCV: Hepatitis C virus; PSM: Propensity score matching

With a view to evaluating the impact of RFA and surgery on OS and DFS, we further divided the Control and CKD groups into RFA and surgery groups. The preoperative characteristics of these two groups are shown in Table 2. In the Control groups, BCLC stage was significantly advanced in the OP subgroup (66% vs. 77%, *p* = 0.038). The Control OP subgroups also had a higher proportion of patients with elevated alpha-fetoprotein level (13% vs. 29%, *p* = 0.005) and HCV carriers (49% vs. 33%, *p* = 0.007). However, the RFA subgroup had a more advance Child–Pugh class (Class B: 5% vs. 0%, *p* = 0.004) and more HBV carriers (40% vs. 58%, *p* = 0.003) than the OP subgroup.

**Table 2:** Baseline characteristics of Control and CKD hepatocellular carcinoma patients

Variables	Control			CKD		
	RFA (n = 134)	OP (n = 147)	*p*-value	RFA (n = 69)	OP (n = 33)	*p*-value
Sex, female, n (%)	50 (37%)	40 (27%)	0.070	34 (49%)	10 (30%)	0.072
Age, year (mean ± SD)	64.54 (10.18)	63.50 (11.14)	0.410	71.33 (8.74)	67.97 (10.80)	0.124
BMI, kg/m^2^ (mean ± SD)	24.8 (4.83)	25.0 (4.08)	0.732	25.6 (3.67)	25.7 (3.90)	0.916
Tumor size (mm)	28.8 (8.8)	29.7 (16.55)	0.907	22.97 (7.78)	31.45 (14.90)	0.004*
BCLC stage, A, n (%)	88 (66%)	113 (77%)	0.038*	50 (72%)	29 (8%)	0.083
Alcohol consumption	44 (33%)	43 (29%)	0.441	9 (13%)	6 (18%)	0.521
ECOG PS, n (%)						
0	131 (97.7%)	143 (97.3%)	0.796	67 (97.1%)	27 (81.8%)	0.007*
1	1 (0.8%)	4 (2.7%)	0.212	2 (2.9%)	6 (18.2%)	0.007*
≥2	2 (1.5%)	0	0.138	0	0	**-**
AFP > 20 ng/ml, n (%)	17 (13%)	38 (29%)	0.005*	11 (16%)	4 (12%)	0.601
Child–Pugh Class, n (%)						
A	126 (94%)	147 (100%)	0.003*	65 (94%)	32 (97%)	0.549
B	8 (5%)	0	0.004*	4 (6%)	1 (3%)	0.549
HBV, n (%)	54 (40%)	85 (58%)	0.003*	23 (33%)	11 (33%)	1
HCV, n (%)	50 (37%)	43 (29%)	0.153	32 (46%)	17 (52%)	0.633
HBV&HCV co-infection, n (%)	5 (4%)	7 (5%)	0.910	3 (4%)	3 (9%)	0.406

BMI: Body mass index; ECOG PS: Eastern Cooperative Oncology Group performance status; AFP: Alpha-fetoprotein; HBV: Hepatitis B virus; HCV: Hepatitis C virus; PSM: Propensity score matching

The pre-treatment characteristics of the RFA and OP subgroups in the CKD group are shown in Table 2. In the CKD group, tumor size was much bigger in the OP subgroup (22.97 mm vs. 31.45 mm, *p* = 0.004). The OP subgroup also had an inferior performance status (Eastern Cooperative Oncology Group Performance Status 0: 97.1% vs. 81.8%, *p* = 0.007). Other tumor factors and liver functions showed no significant difference.

*Advantages of preserved renal function are reduced in overall survival after PSM and have no effect on disease-free survival*.

**Fig. 2:**
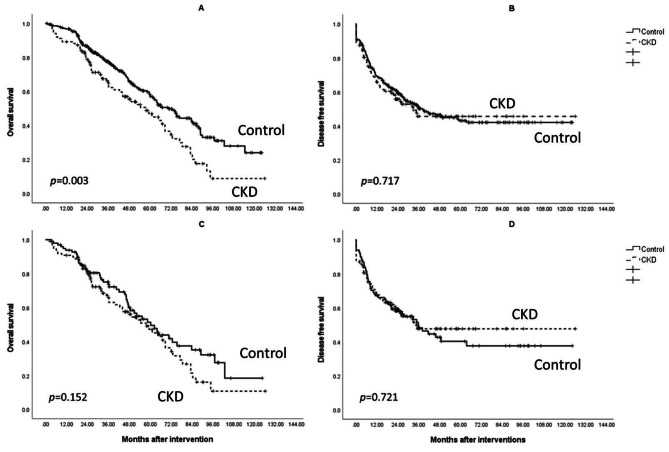
Kaplan–Meier survival analysis of Control and CKD group. (A) Overall survival was significantly better in the Control group. (B) There were no significant differences between the Control group and CKD group in terms of disease-free survival. (C) After propensity score matching, the survival benefit in the Control group faded. (D) There were no significant differences between the Control group and the CKD group in terms of disease-free survival after propensity score matching

The OS and DFS rates of patients with Control and CKD are compared in Fig. 2. The Control group had significantly better OS than the CKD group (72.6 months vs. 56.7 months, *p* = 0.003). DFS was similar between these groups (62.2 months vs. 63.8 months, p = 0.717). However, there was no significant difference in OS between the groups after PSM reduced the age differences (66.6 months vs. 57.6 months *p* = 0.152). DFS consistently showed no significant differences after PSM, similar to before PSM (58.2 months vs. 66.3 months, *p* = 0.721).

### OP consistently results in superior OS in both control and CKD groups

**Fig. 3:**
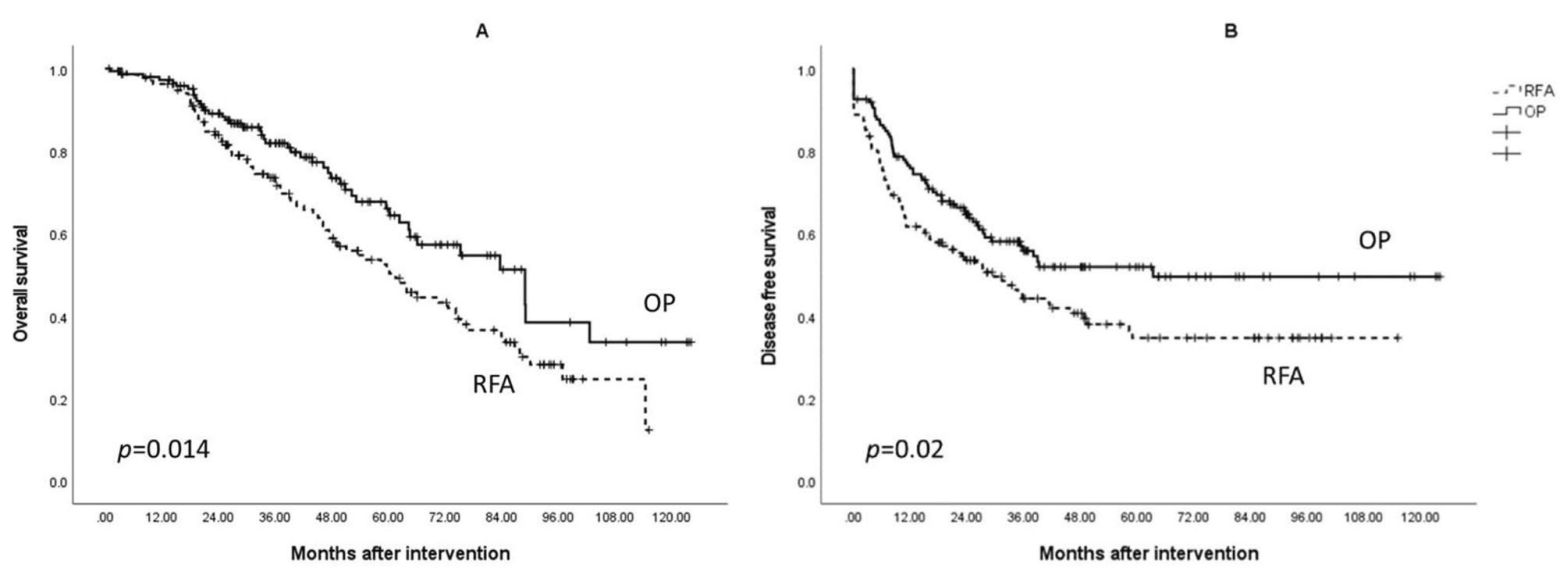
Kaplan–Meier survival analysis of the radiofrequency ablation (RFA) and surgery subgroups in the Control group. (A) Overall survival was significantly better in the OP subgroup. (B) Disease-free survival was significantly better in the OP group

**Fig. 4:**
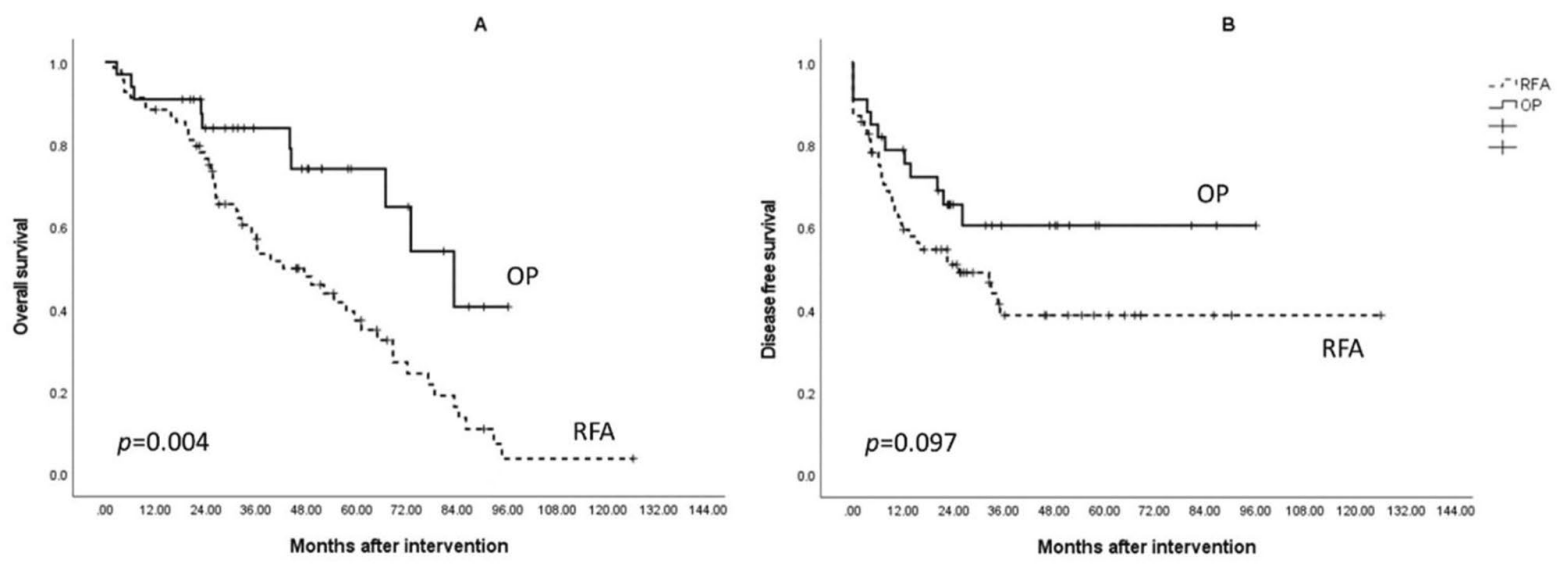
Kaplan–Meier survival analysis of radiofrequency ablation (RFA) and surgery subgroups in the CKD group. (A) Overall survival was significantly better in the OP subgroup. (B) There was no significant difference in disease-free survival between the RFA and OP subgroups

The OS and DFS rates of the patients who underwent RFA and surgery in the Control group are shown in Fig. 3. The OP subgroup had a significantly superior OS (65.0 months vs. 80.0 months, *p* = 0.014) and DFS (50.9 months vs. 70.2 months, *p* = 0.020) as compared to the RFA group. In the CKD group, showed in Fig. 4, the OP group showed a consistent survival advantage in terms of OS (70.6 months vs. 49.2 months, *p* = 0.004), but there was no significant difference in DFS (56.0 months vs. 62.2 months, *p* = 0.097).

Cox proportional hazard model was performed in subgroup analyze (Tables 3 and 4). The results were consistency with previous study and showing operation was a good prognostic factor for OS in both group and for DFS in Control group (control group OS p-value: 0.007, CKD group OS p-value < 0.001, Control group DFS p-value: 0.017). On the other hand, age was considered a risk factor for tumor recur and poor prognosis in CKD patient with HR 1.035 and 1.042 in OS and DFS respectively.

**Table 3:** Cox proportional hazards analyses of prognostic factors for overall survival in Control group and CKD group

Risk factor	Control		CKD	
	Hazard ratio(95%CI)	*p*-value	Hazard ratio(95%CI)	*p*-value
RFA vs. OP	0.558	0.007*	0.207	< 0.001*
Sex, female	0.872	0.555	0.723	0.364
Age	1.016	0.072	1.035	0.046*
BMI	0.976	0.258	1.000	0.995
Tumor size	1.018	0.003*	1.024	0.059
BCLC stage A	1.602	0.55	1.126	0.734
Alcohol consumption	1.509	0.58	1.492	0.393
ECOG PS,0	1.155	0.888	0.379	0.127
AFP > 20 ng/ml	0.803	0.392	2.137	0.061
Child–Pugh Class, A	0.848	0.714	0.685	0.513
HBV	0.784	0.326	0.891	0.785
HCV	0.792	0.370	1.784	0.117
HBV&HCV co-infection	2.045	0.196	0.210	0.084

**Table 4:** Cox proportional hazards analyses of prognostic factors for Disease free survival in Control group and CKD group

Risk factor	Control		CKD	
	Hazard ratio(95%CI)	*p*-value	Hazard ratio(95%CI)	*p*-value
RFA vs. OP	0.637	0.017*	0.577	0.158
Sex, female	1.175	0.434	0.807	0.558
Age	0.998	0.831	1.042	0.020*
BMI	0.973	0.170	0.998	0.957
Tumor size	1.012	0.051	1.002	0.913
BCLC stage A	1.813	0.007*	1.008	0.983
Alcohol consumption	1.258	0.261	0.941	0.914
ECOG PS,0	1.395	0.646	0.563	0.329
AFP > 20 ng/ml	0.869	0.529	1.315	0.521
Child–Pugh Class, A	0.698	0.416	0.566	0.444
HBV	1.045	0.855	0.540	0.540
HCV	1.392	1.392	0.606	0.606
HBV&HCV co-infection	0.646	0.646	0.879	0.879

## Discussion

In this study, we showed that patients with early HCC and CKD in different stages who underwent curative treatments had similar survival outcomes after PSM. Patients with Control had a superior OS outcome as compared to patients with CKD before PSM, while there was no significant difference in the DFS.

Our initial data comparing OS and DFS between Control and CKD among BCLC 0&A HCC patients was similar to those of previous studies that concluded that CKD was a poor prognostic factor in HCC patients. Liu et al. reported that CKD was not only a poor prognostic factor but was also a significant factor for post-operative complications for patients who underwent hepatectomy as treatment [14]. Furthermore, Lee et al. demonstrated that HCC patients with CKD stages 4 and 5 had inferior survival compared to those with CKD stages 1 and 2 [15]. CKD and end-stage renal disease have long been considered to be risk factors for HCC. Several hypotheses have been proposed regarding this relationship, including a dysregulated immune system, defective DNA repair mechanism, and impaired antioxidant defense; however, further evidence is required to prove these links [7, 16].

Previous studies were not filtered by the HCC stage. We assumed that CKD had more of an impact on survival in patients being treated for moderate and advanced HCCs. We found few reports that focused on CKD in patients with early-stage HCC. These patients should undergo curative treatments to potentially have a longer survival outcome. When we studied the baseline characteristic of these patients in our study, we noticed that the CKD group was significantly older than the Control group (Control to CKD: 64.00 vs. 70.25, *p* < 0.001). It is natural that eGFR declines with age. The process is believed to be related to multiple factors, including systemic disease and environmental factors.

Interestingly, after we minimized the age difference between the two groups by PSM, the OS was no longer significantly different between the groups (*p* = 0.152). This result was similar to that of Yoshikawa et al., which showed no significant difference between groups separated by a cutoff eGFR value of 45 ml/min/1.73^2^. However, their study only included patients undergoing hepatectomy [12]. Thus, a CKD bias in HCC patients might be due to age and result in differences in OS. Taiwan had a high prevalence of CKD stages 3–5 (9.06%), with age, male sex, and systemic diseases (such as diabetes mellitus, metabolic syndrome, and hypertension) being significant risk factors [17, 18]. This would had an impact on survival.

We further studied the impact of surgery and RFA on the different CKD groups and found that surgery provided a superior OS and DFS in patients with preserved renal function. These data suggested that, in the Control group, liver resection is the standard treatment option for early-stage HCC if the surgical risk is tolerable [5, 19]. DFS also benefited from surgical resection, with clearer and safer margins, resulting in a superior outcome.

In the CKD group, the OS was consistently superior in the OP subgroup (*p* = 0.004), even though ECOG and tumor size were more unfavorable in the OP subgroup. However, the advantage in terms of DFS vanished in advanced CKD patients. It is well-established that patients with impaired renal function have an increased cancer risk. Their pathophysiology might contribute to the accumulation of uremic toxins, which promote the development of liver fibrosis and cirrhosis [20, 21]. High inflammation status caused by impaired renal function might also play a critical role in the high recurrence rate. Renal dysfunction has been proven to increase the cytokines level in our body, including interleukin [19]-1, IL-6, and tumor necrosis factor-α [22]. These cytokines are associated with HCC risk by accumulating errors and mutations in the cell cycle during necrosis and regeneration, giving hepatocytes more potential for carcinogenesis [23]. The DFS results could also be related to a higher ratio of HBV infection in the OP subgroup, rather than to uremic toxins, as mentioned by Hwang mentioned in his research [24]. The molecular pathways need to be identified clearly, and larger samples, without bias, need to be evaluated in future studies.

Meanwhile, the result of cox proportional hazard model once again confirmed the advantage of operation in each group, especially in the survival benefit for CKD patient with HR: 0.207. It also found that age was a poor prognostic factor for overall survival (OS) and disease-free survival (DFS) in the CKD group. This may be due to the fact that older patients with CKD are more vulnerable to hyper inflammation and uremic toxins. It’s important to note that this result may be influenced by other factors that were not included in the model, and it’s always important to carefully interpret and consider the limitations of any statistical model.

In our study, the results indicated that CKD should not be considered as a poor prognostic factor, which deprived early HCC patients from curative treatment. Our data suggested that liver resection for patients with early HCC and CKD at different stages could obtain a better survival. Thus, our data imply that we should no longer consider CKD as a poor prognostic factor in HCC patients, as the prior conclusion might have been due to the confounding effect of age on CKD.

### Limitations

This was a small sample-size study involving fewer than 400 patients from a single center. Although the size limited the strength of this study, a specialized liver team and tumor board could minimize the associate bias. The comparison between the OP and RFA subgroups within the Control group reveals worse liver function (Child–Pugh class) in the RFA group but a more advanced tumor stage (BCLC) in the surgical subgroup, which could be a bias in our study. We could only divide the patient into two groups due to the sample size. Further studies should be performed to divide CKD patients into more groups with a higher sample size.

## Conclusion

CKD should not be considered a poor prognostic factor in early HCC patients scheduled to undergo curative treatments. Moreover, we should encourage CKD patient to received hepatectomy for a better prognosis base on our study.

## Data Availability

The datasets used during the current study are available from the corresponding author on reasonable request.

## References

[1] Rawla P, Sunkara T, Muralidharan P, Raj JP (2018). Update in global trends and aetiology of hepatocellular carcinoma. Contemp Oncol (Pozn).

[2] McGlynn KA, Petrick JL, El-Serag HB (2021). Epidemiology of Hepatocellular Carcinoma. Hepatology.

[3] Ghouri YA, Mian I, Rowe JH (2017). Review of hepatocellular carcinoma: epidemiology, etiology, and carcinogenesis. J Carcinog.

[4] Reig M, Forner A, Rimola J, Ferrer-Fabrega J, Burrel M, Garcia-Criado A, Kelley RK, Galle PR, Mazzaferro V, Salem R (2022). BCLC strategy for prognosis prediction and treatment recommendation: the 2022 update. J Hepatol.

[5] Llovet JM, Bru C, Bruix J (1999). Prognosis of hepatocellular carcinoma: the BCLC staging classification. Semin Liver Dis.

[6] Tandon P, Garcia-Tsao G (2009). Prognostic indicators in hepatocellular carcinoma: a systematic review of 72 studies. Liver Int.

[7] Yeh H, Chiang CC, Yen TH (2021). Hepatocellular carcinoma in patients with renal dysfunction: pathophysiology, prognosis, and treatment challenges. World J Gastroenterol.

[8] Orii T, Takayama T, Haga I, Fukumori T, Amada N (2008). Efficacy of a liver resection for hepatocellular carcinoma in patients with chronic renal failure. Surg Today.

[9] Toshima T, Shirabe K, Yoshiya S, Muto J, Ikegami T, Yoshizumi T, Maehara Y (2012). Outcome of hepatectomy for hepatocellular carcinoma in patients with renal dysfunction. HPB (Oxford).

[10] Chen YC, Su YC, Li CY, Wu CP, Lee MS (2015). A nationwide cohort study suggests chronic hepatitis B virus infection increases the risk of end-stage renal disease among patients in Taiwan. Kidney Int.

[11] Chen YC, Lin HY, Li CY, Lee MS, Su YC (2014). A nationwide cohort study suggests that hepatitis C virus infection is associated with increased risk of chronic kidney disease. Kidney Int.

[12] Yoshikawa T, Nomi T, Hokuto D, Kamitani N, Matsuo Y, Sho M (2021). Outcomes in patients with chronic kidney Disease after Liver Resection for Hepatocellular Carcinoma. World J Surg.

[13] Levey AS, Eckardt KU, Tsukamoto Y, Levin A, Coresh J, Rossert J, De Zeeuw D, Hostetter TH, Lameire N, Eknoyan G (2005). Definition and classification of chronic kidney disease: a position statement from kidney disease: improving global outcomes (KDIGO). Kidney Int.

[14] Liu X-Y, Zhao Z-Q, Cheng Y-X, Tao W, Yuan C, Zhang B, Wang C-Y. Does Chronic Kidney Disease Really Affect the Complications and Prognosis After Liver Resection for Hepatocellular Carcinoma? A Meta-Analysis. Frontiers in Surgery. 2022;9. 10.3389/fsurg.2022.870946 PMC9019129 35465427

[15] Lee CH, Hsieh SY, Lin JL, Liu MS, Yen TH (2013). Hepatocellular carcinoma in patients with chronic kidney disease. World J Gastroenterol.

[16] Lin HF, Li YH, Wang CH, Chou CL, Kuo DJ, Fang TC (2012). Increased risk of cancer in chronic dialysis patients: a population-based cohort study in Taiwan. Nephrol Dial Transplant.

[17] Tonelli M, Riella M (2014). Chronic kidney disease and the aging population. Int J Organ Transplant Med.

[18] Tsai MH, Hsu CY, Lin MY, Yen MF, Chen HH, Chiu YH, Hwang SJ (2018). Incidence, prevalence, and duration of chronic kidney disease in Taiwan: results from a community-based screening program of 106,094 individuals. Nephron.

[19] Tsilimigras DI, Bagante F, Sahara K, Moris D, Hyer JM, Wu L, Ratti F, Marques HP, Soubrane O, Paredes AZ (2019). Prognosis after resection of Barcelona Clinic Liver Cancer (BCLC) Stage 0, a, and B Hepatocellular Carcinoma: a Comprehensive Assessment of the current BCLC classification. Ann Surg Oncol.

[20] Sun CY, Chang SC, Wu MS (2012). Uremic toxins induce kidney fibrosis by activating intrarenal renin-angiotensin-aldosterone system associated epithelial-to-mesenchymal transition. PLoS ONE.

[21] Nitta T, Kim JS, Mohuczy D, Behrns KE (2008). Murine cirrhosis induces hepatocyte epithelial mesenchymal transition and alterations in survival signaling pathways. Hepatology.

[22] Ebert T, Neytchev O, Witasp A, Kublickiene K, Stenvinkel P, Shiels PG (2021). Inflammation and oxidative stress in chronic kidney Disease and Dialysis Patients. Antioxid Redox Signal.

[23] Rico Montanari N, Anugwom CM, Boonstra A, Debes JD. The Role of Cytokines in the Different Stages of Hepatocellular Carcinoma. Cancers (Basel). 2021;13(19). 10.3390/cancers13194876 PMC8508513 34638361

[24] Hwang JC, Weng SF, Weng RH (2012). High incidence of hepatocellular carcinoma in ESRD patients: caused by high hepatitis rate or ‘uremia’? A population-based study. Jpn J Clin Oncol.

